# Recurrent Germline Mutations of CHEK2 as a New Susceptibility Gene in Patients with Pheochromocytomas and Paragangliomas

**DOI:** 10.1155/2021/1392386

**Published:** 2021-09-30

**Authors:** Yinjie Gao, Chao Ling, Xiaosen Ma, Huiping Wang, Yunying Cui, Min Nie, Anli Tong

**Affiliations:** ^1^NHC Key Laboratory of Endocrinology (Peking Union Medical College Hospital), Department of Endocrinology, Peking Union Medical College Hospital, Peking Union Medical College, Chinese Academy of Medical Sciences, Beijing 100730, China; ^2^Laboratory of Clinical Genetics (Peking Union Medical College Hospital), Peking Union Medical College Hospital, Peking Union Medical College, Chinese Academy of Medical Sciences, Beijing 100730, China

## Abstract

**Purpose:**

Recently, pheochromocytomas and paragangliomas (PPGLs) have been strongly suspected as hereditary tumors, as approximately 40% of patients carry germline mutations. In the cancers where defects occur to corrupt DNA repair and facilitate tumorigenesis, a *CHEK2* strong association has been observed. Therefore, the purpose of this study was to investigate the effect of *CHEK2* mutations for its possible pathogenicity in PPGLs.

**Methods:**

Four patients with *CHEK2* mutations were recruited, as previously detected by the whole exome sequencing. Sanger sequencing was used to verify the germline mutations as well as the loss of heterozygosities (LOHs) in their somatic DNAs. Immunohistochemistry was used to analyze the expression of CHEK2 and its downstream target p53 Ser20 (phosphorylated p53).

**Results:**

The average age of studied patients was 44.25 ± 11.18 years, at the time diagnosis. One patient had multiple tumors which recurred quickly, while two patients had distant metastasis. None of the patient had any relevant family history. Four germline *CHEK2* mutations were identified (c.246_260del; c.715G > A; c.1008+3A > T*;* and c.1111C > T). All the patients were predicted to have either pathogenic or suspected pathogenic mutations. There was no LOH of *CHEK2* gene in somatic DNAs found. Additionally, neither CHEK2 proteins nor its downstream target p53 Ser20 were expressed in the tumor tissues. The inactivation of CHEK2 leads to the decrease in the p53 phosphorylation, which might promote tumorigenesis.

**Conclusions:**

For the first time, *CHEK2* was identified as a susceptibility gene for PPGLs. However, the penetrance of *CHEK2* gene with genotype-phenotype correlation needs to be investigated.

## 1. Introduction

The neuroendocrine tumors arising in the chromaffin cells of adrenal medulla are termed as pheochromocytomas (PCCs), whereas the extra-adrenal tumors originating in the chromaffin cells from the sympathetic and parasympathetic ganglia are known as paragangliomas (PGLs) [1]. PCCs and PGLs (PPGLs) affects around 2–5 patients/million/year, with the prevalence of about 1/300000 to 1/100000 for general population [2]. In the recent years, molecular pathogenesis of this group of lesions has advanced significantly. Almost 40% of PPGLs patients carry germline mutations in a growing list of genes including SDHA, SDHB, SDHC, SDHD, SDHAF2, VHL, RET, MAX, TEMEM127, FH, NF1, and KIF1B [3, 4]. Besides, genes such as EGLN1, EGLN2, MDH2, SLC25A11, *MERTK*, *DLST*, and *KMT2D* are also shown to be related to PPGLs [5–10]. It is noteworthy that the majority of individuals with clinical features such as family history of PPGLs, multiple tumors, and an early age of onset might be indicative of a hereditary onset, but they lack mutations in any of the known PPGLs susceptibility genes.

Cell cycle checkpoint kinase 2 (*CHEK2*) is located in chromosome 22q12.1, which encodes multifunctional kinase crucial for cell cycle regulation, DNA repair, and apoptosis [11]. In response to DNA damage, CHEK2 is required for bridging between ataxia telangiectasia mutated (ATM) kinase with its downstream checkpoint effectors; therefore, CHEK2-deficient patients may have corrupt DNA repair and conserved mutations which ultimately facilitate tumorigenesis [12]. However, as candidate tumor suppressor, CHEK2 contributes to molecular pathogenesis in various human malignancy. Thereby, heterozygous *CHEK2* gene germline mutations have been observed in patients with the Li-Fraumeni cancer-predisposition syndrome (LFS), with other cancers such as breast cancer, colon cancer, thyroid cancer, bladder cancer, ovarian cancer, gastric cancer, renal cancer, and prostate cancer [13]. Hence, *CHEK2* is speculated to be a low-penetrance, multiorgan cancer susceptibility gene.

Recently, whole exome sequencing (WES) technology has been employed to detect germline variations of 121 patients who did not have mutations on definite pathogenic genes. In our previous report, the use of next-generation sequencing (NGS) covering SDHA, SDHB, SDHC, SDHD, SDHAF2, VHL, RET, MAX, TEMEM127, FH, NF1, and KIF1B was analyzed in the cohort with 314 PPGL patients [3]. Among them, four patients showed *CHEK2* gene heterozygous mutations. However, definitive validation of *CHEK2* gene was required to ascertain it as a new candidate susceptibility gene in PPGLs and for the potential value for genetic risk assessment, prognosis, and surveillance. Therefore, this present study aims to investigate the effect of *CHEK2* mutations on DNA-damage pathway and to assess its possible pathogenicity in PPGLs.

## 2. Materials and Methods

### 2.1. Patients

Out of PPGLs cohort, four patients (Patients 1, 2, 3, and 4) had variants of *CHEK2* gene as detected by the WES. The PPGLs cohort was recruited from the Peking Union Medical College Hospital between November 2007 and June 2013 (the detailed data of all 121 patients who received WES are not provided in this study). The collected blood samples and formalin fixed paraffin embedded (FFPE) tumor tissues and sections were collected after obtaining the written informed consent from the patients. The approval of the study was granted by the medical ethics committee of the hospital, and the results of this research were also agreed to be published. The DNAs from the peripheral blood leukocytes (Omega Blood DNA Midi Kit, Omega Bio-Tek, USA) and FFPE tumor tissues (Quick-DNATM FFPE Kit, ZYMO RESEARCH, USA) were obtained using a standard procedure from the patients having *CHEK2* mutations.

### 2.2. Sanger Sequencing of *CHEK2* Gene

The four mutations of *CHEK2* gene detected by WES were verified by the PCR amplification in combination with Sanger sequencing. The PCR primers and amplification methods are shown in Table 1. For distinguishing the sequence of *CHEK2* with the highly homologous pseudogenes (*CHEK2P1-5*) from exon 11 to exon 15, we used nested PCR amplification for detecting mutation on exon 11 of Patient 4. All the sequences were studied for the mutations, by comparing them with the reference sequence of the *CHEK2* gene (NM_007194.4 and NP_009125.1) through the NCBI website.

### 2.3. Loss of Heterozygosity (LOH) of *CHEK2* in Tumor Tissue

PCR amplification and Sanger sequencing were done to evaluate the LOH of corresponding sites in somatic DNAs of patients. However, one patient FFPE sample (Patient 4) was not sufficient; therefore, only three patients FFPE tumor tissues were subjected for studying the corresponding exons of *CHEK2* with mutations in somatic DNA by using the sequencing method mentioned in Table 2. The homozygous mutant for supporting that LOH of CHEK2 was present, and for the heterozygote means, no LOH in the corresponding site in somatic DNA was found.

### 2.4. CHEK2 Immunohistochemistry

The four patients with *CHEK2* mutations were also evaluated for CHEK2 protein expression in the FFPE tumor sections by immunohistochemistry (IHC). Briefly, the sections were incubated with primary antibody of human Anti-Chk2 antibody (ab207446) (Abcam, England) at 1/100 dilution, followed by secondary incubation with the goat anti-rabbit IgG polymer (PV-9001, (ZSGB-BIO, China)) at 1/500 dilution. As a positive control, normal gland and PPGL tumor tissue with *RET* mutation were used.

### 2.5. Immunohistochemistry of Downstream Target p53 Ser20

Further, *CHEK2* downstream target p53 Ser20 (phosphorylated p53 by functional CHEK2) expression was evaluated by IHC. Briefly, the sections were incubated with primary antibody of human Anti-p53 Ser20 antibody (ABP50383) (Abbkine, China) at 1/200 dilution, followed by the secondary incubation with goat anti-mouse/rabbit IgG polymer (PV-8000, (ZSGB-BIO, China)) at 1/500 dilution, whereas the sections of normal gland were used as a positive control.

## 3. Results

### 3.1. Clinical Manifestation

The detailed clinical symptoms of the four patients with *CHEK2* mutations are shown in Table 3. Among the 4 patients, three were male and 1 was female patient. The average age of the patients at the time of diagnosis was 44.25 ± 11.18 years old, where Patient 2 was only 30 years old at the time of PPGLs onset. Patient 1 had adrenal and paraaortic multiple tumors, which recurred in situ after surgery. Two patients had distant metastasis (Patient 2: liver metastasis and Patient 4: bone metastasis); however, no patients had family history.

### 3.2. Mutation Sites of *CHEK2*

**Figure 1:**
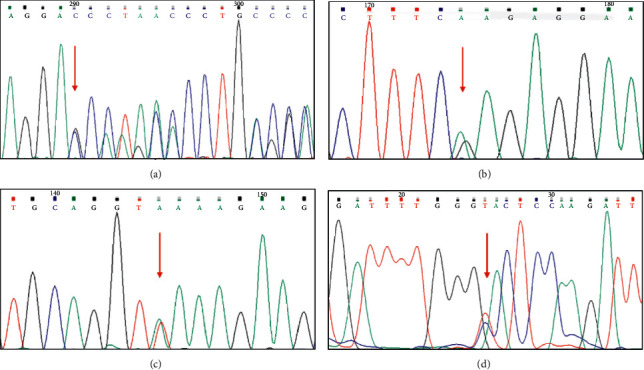
The germline *CHEK2* mutations detected by Sanger sequencing of these four patients. The red arrows indicate the mutation sites, (a) for Patient 1, (b) for Patient 2, (c) for Patient 3, and (d) for Patient 4.

In the studied patients, four *CHEK2* germline mutations were detected, including two missenses (c.715G > A, p.E239K and c.1111C > T, p.H371Y), one deletion (c.246_260del, p.82_87del (<50 bp)), and one splice site mutation (c.1008+3A > T). The results of Sanger sequencing are shown in Figure 1. The American College of Medical Genetics (ACMG) guidelines were used to predict the pathogenicity of the detected four variants. Two of the variants were evaluated as pathogenic mutations (Patient 2: c.715G > A, p.E239K; Patient 4: c.1111C > T, p.H317Y), and the other two were as suspected pathogenic mutations (Patient 1: c.246_260del, p.82_87del; Patient 3: c.1008+3A > T). The detailed information about the detected mutations and ACMG evaluations are shown in Table 4. Of note, these four patients had no other germline mutations of the confirmed susceptibility genes for PPGLs.

The evidences of pathogenicity of ACMG mentioned in this table were as follows: PS1: the same amino acid change as a previously established pathogenic variant regardless of nucleotide change; PS3: well-established in vitro or in vivo functional studies supportive of a damaging effect on the gene or gene product; PM1: located in a mutational hot spot and/or critical and well-established functional domain (e.g., active site of an enzyme) without benign variation; PM2: absent from controls (or at extremely low frequency if recessive) in Exome Sequencing Project, 1000 Genomes Project, or Exome Aggregation Consortium; PM4: protein length changes as a result of in-frame deletions/insertions in a nonrepeat region or stop-loss variants; PM6: assumed de novo, but without confirmation of paternity and maternity; PP3: multiple lines of computational evidence support a deleterious effect on the gene or gene product (conservation, evolutionary, splicing impact, etc.); PP5: reputable source recently reports variant as pathogenic, but the evidence is not available to the laboratory to perform an independent evaluation.

### 3.3. LOH of *CHEK2* in Tumor Tissue

**Figure 2:**
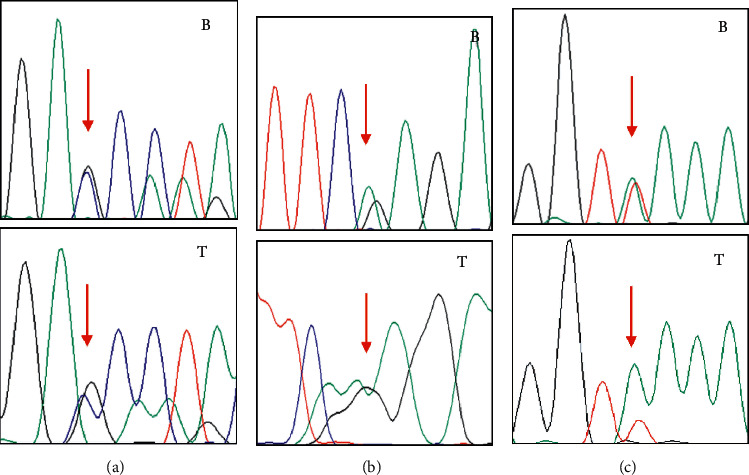
The somatic CHEK2 mutations detected of three patients compared with the germline mutation sites. The other one patient' sufficient FFPE sample was not obtained. The red arrows indicate the mutation sites, (a) for Patient 1, (b) for Patient 2, and (c) for Patient 3. “B” means the germline sites from peripheral blood leukocytes, and “T” means the somatic sites from FFPE tumor tissues.

In the FFPE tumor tissues, the sites of three mutations detected in peripheral blood DNA were heterozygous in somatic DNAs (Note: Patient 4 had insufficient FFPE tumor tissues) (Figure 2). The results confirm that there was no LOH of *CHEK2* gene in the studied patients.

### 3.4. Immunohistochemistry of CHEK2 Protein and the Downstream Target p53 Ser20

**Figure 3:**
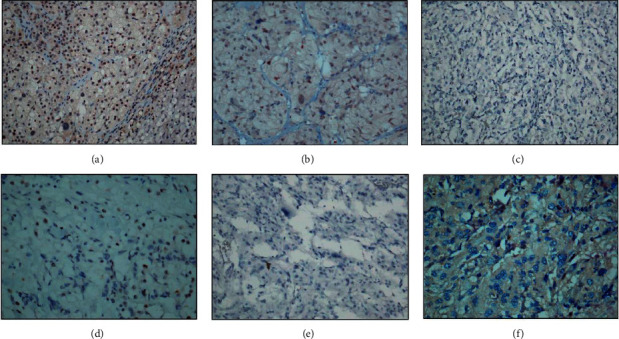
Immunohistochemical staining of CHEK2 protein. (a) Staining for normal gland (positive control: the nucleus was positive for CHEK2). (b) Staining for PPGL tumor tissue with *RET* mutation (positive control: the nucleus was positive for CHEK2), (c) for Patient 1 (negative for CHEK2), (d) for Patient 2 (negative for CHEK2), (e) for Patient 3 (negative for CHEK2), and (f) for Patient 4 (the nucleus was negative but partial cytoplasm was weak positive for CHEK2).

**Figure 4:**
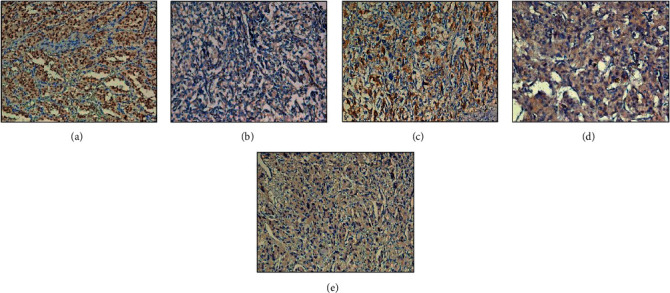
Immunohistochemical staining of the phosphorylated p53 Ser20. (a) Staining for normal gland (positive control: the nucleus was positive for p53 Ser20), (b) for Patient 1 (negative for p53 Ser20), (c) for Patient 2 (the nucleus was negative but partial cytoplasm was positive for p53 Ser20), (d) for Patient 3 (negative for p53 Ser20), and (e) for Patient 4 (the nucleus was negative but partial cytoplasm was weak positive for p53 Ser20).

Compared with the normal adrenal or PPGL tumor tissue sections with *RET* mutation (the nucleus was positive for CHEK2 staining), the results of CHEK2 immunohistochemistry were negative in all patients except that the partial cytoplasm was weakly positive for Patient 4. This finding suggested that the CHEK2 proteins were either not expressing or inactivated in the tumor tissues (Figure 3). The results of the downstream target p53 Ser20 immunohistochemical staining were nucleus negative for these patients (except for partial cytoplasm positivity in Patients 2 and 4), as compared with positive control from normal gland tissue. These findings further confirm that the inactivation of CHEK2 could result in the decrease activity of phosphorylation of p53 protein (Figure 4). Therefore, the abnormal phosphorylation of p53 protein might influence the biological function and can lead to tumorigenesis.

## 4. Discussion

We previously shown that the *CHEK2* gene mutations accounted for 3.3% (4/121) of PPGLs patients, in which pathogenic mutations of the related genes were not detected, whereas in 1.3% (4/314) of PPGLs patients recruited cohort from Peking Union Medical College Hospital, a frequency equivalent to a few identified PPGLs susceptibility genes including *SDHA*, *TMEM127*, *MAX*, and *FH* was found [14–17]. It is noteworthy that *CHEK2* gene mutations might be associated with the genetic background of PPGLS, as out of 4, three patients detected *CHEK2* mutations were presented with the multiple tumors or malignant developments.

**Figure 5:**
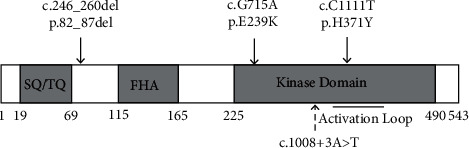
The pattern diagram of functional domain on *CHEK2* gene and the location of the four detected mutations.

Since checkpoint defects result in the accumulation of altered genetic information and a central feature of carcinogenesis, these DNA-damage checkpoint pathways have been of interest to the field of cancer biology [18]. Among the conserved DNA-damage activated kinases identified so far, the CHEK2 plays a central role in implementing many aspects of the checkpoint response, related to the occurrence of various cancers [19]. The CHEK2 protein contains three distinct functional domains: (1) the SQ/TQ-rich, (2) the forkhead-associated, and (3) and the serine/threonine kinase domain [20].  Figure 5 shows the pattern of *CHEK2* gene and the four detected mutations location. However, except for the one mutation which was present next to the SQ/TQ-rich domain, all others were in the kinase domain.

The detected four germline variants of *CHEK2* in this study were causing decreased expression of the CHEK2 protein, suggesting the alterations were resulting in loss-of-function pathogenicity. Though in PPGLs, the function of *CHEK2* gene has not been well characterized; however, *CHEK2* role in cell proliferation and tumor suppression has been confirmed by various reports. Hong et al. established a CHEK2-1100delC mutant, which promoted the gastric cancer cell proliferation, migration, and invasion, with downregulation of E-cadherin and upregulated vimentin expression, suggesting its possible role in altered biological behavior as epithelial mesenchymal transition (EMT) [21]. Another study reported the novel recurrent CHEK2-Y390 C mutant associated with increased breast cancer risk in Chinese population. The study further reported that the mutant protein's inability resulted in the lack of phosphorylation of CDC25 A Ser178 and p53 Ser20 after DNA damage, which was led to abnormal cell apoptosis and checkpoint repair [22]. In the present study, we also found that the p53 could not be phosphorylated due to *CHEK2* mutations in the studied four patients, indicating the inability of variant CHEK2 proteins to efficiently bind and phosphorylate its substrates.

Among these four mutations found in the present study, two missense mutations were reported previously. In year 2003, the mutant CHEK2-E239 K was first mentioned for the prostate cancer [23]. The alteration of amino acid in the kinase activation domain significantly alter the phosphorylation of p53 in DNA-damage signaling, while the wild type CHEK2 completely retained CHEK2 kinase activity following ionizing radiation, and only 50% response was regained in the mutant group [24]. This studied mutation was later detected in patients with breast cancer and non-Hodgkin's lymphoma [25, 26]. Another mutant CHEK2-H371Y detected in our study was confirmed as a breast cancer risk variant in 2011, for 4% of the total patients. Approximately 50% decrease was observed during functional analysis for the autophosphorylation, transphosphorylation, and *CHEK2* activity of CHEK2-H371Y mutant [27]. The other two variants namely p.82_87del and c.1008 + 3A > *T* detected in our study were not reported in databases previously. Both had high pathogenicity as evaluated by the ACMG, suggesting that these *CHEK2* mutations could be deleterious as they might influence the protein structure and kinase domain. Additionally, no LOHs were detected in these corresponding sites of the studied four patients with *CHEK2* mutations. Moreover, haploinsufficiency caused by dominant negative effect, or the change in protein spatial structure with the mutant amino acid folding, can lead to the abnormal function by only one allele variant [28, 29].

In the present study, lack of family history in four pedigrees was investigated for genes such as *MDH2*, *BAP1*, *DLST,* or *SLC25A11* [6, 9, 10, 30]. However, among the de novo mutation or low-penetrance inheritance, the latter is frequently associated with PPGLs [6, 31]. On the other hand, due to the advancement in genetics, germline mutations and familial syndromes are known to be associated with 8–24% of sporadic PPGLs [2]. Germline testing is now generally recommended in PPGL, and besides the potential role played in PPGLs pathogenesis, the detection of germline variants in patients clinically defined as sporadic may be helpful in finding out the existence of unknown multineoplasia hereditary diseases [32, 33].

The current study had the following limitations. First, due to the limitation of follow-up year, we did not observe the other multiple tumors in these patients with *CHEK2* mutations or their family members. Second, the DNAs from blood leukocytes of patient's parents were not obtained; therefore, we could not identify if the mutations had de novo origin. Thirdly, all *CHEK2* variants detected in somatic DNA were heterozygous; therefore, the potential mechanisms leading to the abnormal function by only one allele variant should be further researched. Lastly, in the results of IHC for CHEK2 expression, tumor or normal adrenal tissue, stromal cells, such as vascular endothelial cells, were not stained positive. These findings on CHEK2 staining were also discussed in previous studies [34–37]. Therefore only positive or negative staining of tumor cells was compared and analyzed here.

## 5. Conclusions

In conclusion, we have identified four germline variants, which functionally compromises *CHEK2*, suggesting *CHEK2* as a susceptibility gene for PPGLs. However, due to the limited number of patients and low prevalence of the *CHEK2* mutations, more cases are required for the validation of its penetrance and genotype-phenotype correlation in PPGLs.

## Figures and Tables

**Table 1:** PCR primers for four CHEK2 germline mutations.

Primer	Upstream	Downstream
Exon 2	ACTTTTTAATTTTAAGTCTTG	AACGTGCCAAAAACCTGGAC
Exon 6	GCCCTTGACATTTTACACT	CAAATTCATCCATCTAAGCAGG
Intron 9	TTGTTTTATTGTCTTCTGTCCAA	TTTTAATCCACGGTCCCTC
Nested PCR		
Exon 11–15	CGACGGCCAGTCTCAAGAAGAGGACTGTCTT	GCTATGACCATGCACAAAGCCCAGGTTCCATC
Exon 11	GCAAGTTCAACATTATTCCCTTTT	ATCACCTCCTACCAGTCTGTGC

(a) The condition of PCR amplification for Exon 2, 6, and Intron 9 was as follows: predenaturation at 95°C for 5 min, denaturation at 95°C for 30 s, annealing at 54°C/52°C/64°C for 30 s, and extension at 72°C for 40 s. A total of 35 cycles were carried out, final extension at 72°C for 10 min. (b) The condition of nested PCR amplification for Exon 11 was as follows: (1) long-range PCR: predenaturation at 98°C for 5 min, denaturation at 95°C for 30 s, annealing at 68°C for 30 s, and extension at 72°C for 3 min. A total of 35 cycles were carried out, final extension at 72°C for 10 min. Product of long-range PCR was used as a template to amplify the exon 11 using the appropriate oligonucleotide primers. (2) The condition of PCR amplification with the touch-down PCR was as follows: predenaturation at 95°C for 5 min, denaturation at 95°C for 30 s, annealing at 64°C for 1 min (decreased by 0.5°C per cycle), and extension at 72°C for 40 s in 9 cycles, and, next, predenaturation at 95°C for 5 min, denaturation at 95°C for 30 s, annealing at 60°C for 1 min, and extension at 72°C for 40 s in 25 cycles. A total of 34 cycles were carried out, final extension at 72°C for 10 min. (c) PCR products were identified by 1.5% agarose gel electrophoresis and sent to the Beijing SinoGenoMax Company for purification and sequencing. The sequencing was performed by ABI 3730XL instrument.

**Table 2:** PCR primers for CHEK2 mutations in somatic DNA from FFPE tissues.

Primer	Upstream	Downstream
2S300	CACTGAGCTCCTTAGAGAC	CAAGATTGGCAAATCCATC
6S770	TTTGTTTTTCCCTCTAGTGGT	ATTATTTTGGGAAGTTATGAAG
9S41980	GAGCTGTTTGACAAAGTGGT	GTTTTAATCCACGGTCCCT

(a) The condition of PCR amplification was as follows: predenaturation at 95°C for 5 min, denaturation at 95°C for 30 s, annealing at 56°C/52°C/56°C for 30 s, and extension at 72°C for 30 s. A total of 35 cycles were carried out, final extension at 72°C for 10 min. (b) PCR products were identified by 1.5% agarose gel electrophoresis and sent to the Beijing SinoGenoMax Company for purification and sequencing. The sequencing was performed by ABI 3730XL instrument.

**Table 3:** The detailed clinical manifestations of the four patients with CHEK2 mutations.

Patient	Gender	Age at diagnose	Duration	Tumor	NE	E	DA	Past history	Multiple tumors	Tumor recurrence	Tumor metastasis	Family history
1	Male	55	7	PCC, PGL	537.43	3.54	345.60	Renal cyst, cerebral infarction	Adrenal, abdominal	Recurrence	No	No
2	Male	30	13	PCC	714.71	7.15	472.23	No	No	No	Liver	No
3	Male	41	7	PCC	775.03	2.83	571.37	No	No	No	No	No
4	Female	51	5	PCC	1608.66	4.74	342.87	No	No	No	Bone	No

PCC: pheochromocytoma; PGL: paraganglioma; NE: 24-hour urinary norepinephrine (normal range: 16.7–40.7 *μ*g/24 h); E: 24-hour urinary epinephrine (normal range: 1.7–6.4 *μ*g/24 h); DA: 24-hour urinary dopamine (normal range: 120.9–330.6 *μ*g/24 h); NE, E, and DA were the preoperative hormone levels of each patient and measured in *μ*g/24 h. Age at diagnosis and duration of PPGL were measured in years.

**Table 4:** Detailed information of mutations and ACMG evaluation.

Patient	Location	Base change	Amino acid change	ACMG	Pathogenicity
1	Exon 2	c. 246_260del	p. 82_87del	PM2/PM4/PM6	Suspected pathogenic
2	Exon 6	c. G715A	p. E239K	PS1/PS3/PM1/PM6/PP3/PP5	Pathogenic
3	Intron 9	c. 1008+3A > T	—	PM1/PM2/PM6	Suspected pathogenic
4	Exon 11	c. C1111T	p. H371Y	PS3/PM1/PM6/PP3	Pathogenic

## Data Availability

Data would be available upon request from corresponding author of this manuscript.
